# Supplementary material for: Bridging Inequity Gaps in Healthcare Systems While Educating Future Healthcare Professionals—The Social Health Bridge-Building Programme
Source: Int J Environ Res Public Health. 2023 Sep 27;20(19):6837. doi: 10.3390/ijerph20196837 (PMC10572531; doi:10.3390/ijerph20196837)
Supplement: Supplementary file 1 [file ijerph-20-06837-s001.zip › ijerph-2573295-supplementary.pdf]

Supplementary Material S1: The TIDieR checklist

Item 1. Brief name
The Social Health Bridge-Building Programme.

Item 2. Why: describe rational and theory essential to the intervention
Please see element 1-4 in the result section of the paper.

Item 3 What (materials)
The facilities include an office, flyers, teaching resource for health care student volunteers and IT-system (SharePoint). The IT-system contains usable and relevant information about the individuals, which has been obtained with the consent from the individual or someone close to the individual (e.g. relatives, social workers or caregivers). The information is used to create the best assumptions for the bridge-builders before the accompaniment. Furthermore, the IT-system contains a calendar, where the employees have an overview of the accompaniments. Before an accompaniment, an employee informs the bridge-builder by assigning the accompaniment in the IT-system. An Advisory Hotline was established to coordinate appointments between individuals living in vulnerable situations and bridge-builders and to clarify the needs of the individual in relation to the specific health care appointment. The activities are presented in Figure 2 (Logic model).

Item 4 What (procedures and activities)
The core task of the bridge-builder is to accompany individuals living in vulnerable situations to health care appointments. Prior to the meeting with the individual, the bridge-builder assess information about the individuals needs and the type and location of the healthcare appointment. Then the bridge-builder meet with the individual at the agreed location (e.g. the home of the individual address, a nursing facility or shelter). After an initial conversation about the individuals' expectations and needs in relation to the healthcare appointment, the bridge-builder accompany the individual to the health care appointment by taxi, bus or other means of transport. The bridge-builder take part in the consultation with the healthcare professional or stays in the waiting rooms according to the needs of the individual. After the appointment the bridge-builder accompany the individual to their place of residence or another agreed place, discussing the appointment if necessary. If needed and with consent from the individual, information regarding outcome of the health care appointment is distributed to the individuals' carers or relatives. After the accompaniment the bridge-builder contact Social Health's Advisory Hotline and talk about how the appointment went and if there are any issues to be aware of regarding the individual. Employed coordinators are involved in the recruitment, training and supervision of bridge-builders. Furthermore, the coordinators are in charge of coordinating appointments between individuals and bridge-builders (Advisory Hotline). In order to increase awareness and utility of bridge-builders the coordinators lobbies extensively with the community, hospitals, and health educational institutions. An external supervisor provide supervision to bridge-builder and coordinators in order to increase their ability to interact with individuals living in vulnerable situations.

Item 5. Who provided
The team consists of bridge-builders (health care student volunteers), coordinators, supervisors, CEO, user representatives and a national board. The bridge builders are healthcare student volunteers (e.g. future

nurses, medical doctors, phycologists, public health students etc.). The bridge-builders participate in a preparatory course in a total of 20 hours concerning social inequality, communication and boundary setting. In addition, bridge-builders attend in supervision sessions four times a year. During supervision the bridge-builder reflects on the experience-based knowledge and expertise they have learnt from their accompaniments' to healthcare appointments. In the setting of the supervision they act as co-reflectors on each other's experiences. The healthcare student volunteers get further education during arrangements which contains a professional element. The employed coordinators are an interdisciplinary group with various educational background and work experiences especially within the social and health care system. The employees participate in two supervision settings a year which give an opportunity for development and reflection in the work with special focus on the contact to the individuals living in vulnerable situations. The external supervisor is a trained psychologist or psychotherapist. User representatives are individuals living in vulnerable situations who have experience with using bridge-builders in relation to health care appointments. They have been chosen through the social network of Social Health in the light of their set of values. The national board consists of a chair and a vice chair, CEO, a bridge-builder representative, an employee representative and a user representative. The user representative is essential to ensure a perspective of the individuals living in vulnerable situations. Further, the board consists of five individuals with competencies in the field of social inequity, NGO's (e.g. management, strategy and development of NGO's), law, economy and fundraising. There are representatives from various sectors for (e.g. educational institutions, the specialized healthcare system etc.) and an important factor behind the selection of them are their set of values.

Item 6. How: modes of delivery
No preadmission evaluation is needed. Anyone (e.g. individuals, contact persons, healthcare professionals or relatives) can contact the Advisory Hotline if they or someone they know need an accompaniment to an appointment in the healthcare system. The intervention (i.e. accompaniment) is provided individually face-to-face.

Item 7. Where: type of location
The location for the accompaniment depends on the needs of the individual and may differ between the individuals' own house, a shelter, nursing facility or the location of the health care appointment. The means of transportation is agreed upon based on an individual assessment of needs and can for instance include taxi, public transportation, bicycle or walk. The accompaniment is terminated at the individuals' home address or at the given meeting point. The transportation costs of the volunteer are reimbursed by the organization *Social Health*. Recruitment of bridge-builders take place at health education institutions (e.g. medicine, nursing, public health, physical education, psychology, social and health care) through presentations or street canvassing. If possible an employee, a user representative and a bridge-builder visit lectures at the health education institutions to talk about Social Health from different perspectives. Further, posts and advertisement on social medias and website are posted in order to recruit health care students.

Item 8. When and how much
The frequency of accompaniment depends on the needs of the individual and is not confined to a minimum or maximum. Some of the individuals get accompaniment more than once and sometimes with the same healthcare student. The healthcare system defines the time for the appointments and the number and

duration of the appointments. The duration of the accompaniment is not predetermined and depends on the duration of the health care appointment. The bridge-builders are usually each available for appointments two days a month. The recruitment and training of the healthcare students take place once or twice a year. It last for more than three weeks and the preparation of the recruitment last for two weeks.

Item 9. Tailoring
Being flexible in their work with individuals in vulnerable situations is a key issue in the Social Health Bridge-Building Programme because the individuals often meet a difficult and complex system. Therefore, every accompaniment is individually arranged to match the needs and expectations of the individual experiencing vulnerability. In principle, the appointments need to be health-related but if the individual needs help with other problems that prevent them from being able to go to an appointment the Social Health Bridge-Building Programme also help with that. In training of the healthcare student volunteers (the 20-hour course) Social Health are flexible if the students cannot participate due to illness or other factors. In those cases, they facilitate lessons where those who did not participate can get the most necessary content of the training.

Item 10. Modifications during the study
Since the foundation of the Social Health Bridge-Building Programme there have been some amendments. For instance, the healthcare students have been replaced by previous healthcare professionals in some of the smaller cities than the university cities, where the supply of students are minimal.

Item 11. How well (planned)
After every accompaniment the bridge-builder documents the procedure of the accompaniment in the journal system SharePoint. Furthermore, the bridge-builder is briefed by the coordinator before the accompaniment and debriefed afterwards.

Item 12. How well (actual)
Attendance will be reported in future evaluation study.
